# Severe osteomyelitis caused by Myceliophthora thermophila after a pitchfork injury

**DOI:** 10.1186/1476-0711-5-21

**Published:** 2006-09-08

**Authors:** Lauren Destino, Deanna A Sutton, Anna L Helon, Peter L Havens, John G Thometz, Rodney E Willoughby, Michael J Chusid

**Affiliations:** 1 Department of Pediatrics, Medical College of Wisconsin and Children's Hospital of Wisconsin, USA; 2 Fungus Testing Laboratory, Department of Pathology, University of Texas Health Sciences Center, San Antonio, TX, USA; 3 Pharmacy Department, Children's Hospital of Wisconsin, USA; 4 Department of Orthopedic Surgery, Medical College of Wisconsin and Children's Hospital of Wisconsin, USA

## Abstract

**Background:**

Traumatic injuries occurring in agricultural settings are often associated with infections caused by unusual organisms. Such agents may be difficult to isolate, identify, and treat effectively.

**Case report:**

A 4-year-old boy developed an extensive infection of his knee and distal femur following a barnyard pitchfork injury. Ultimately the primary infecting agent was determined to be Myceliophthora thermophila, a thermophilic melanized hyphomycete, rarely associated with human infection, found in animal excreta. Because of resistance to standard antifungal agents including amphotericin B and caspofungin, therapy was instituted with a prolonged course of terbinafine and voriconazole. Voriconazole blood levels demonstrated that the patient required a drug dosage (13.4 mg/kg) several fold greater than that recommended for adults in order to attain therapeutic blood levels.

**Conclusion:**

Unusual pathogens should be sought following traumatic farm injuries. Pharmacokinetic studies may be of critical importance when utilizing antifungal therapy with agents for which little information exists regarding drug metabolism in children.

## 

Myceliophthora thermophila is a thermophilic phaeoid mould found in pasture soil, wood chips, straw, mouldy hay, compost piles and other environmental settings where heat is generated. It is also found in the excreta and rumen of cattle and is a pathogen of cultivated mushrooms [1]. A rare cause of invasive human infections, it can be difficult to isolate and identify in clinical specimens. We recently cared for a 4-1/2 year old boy who developed osteomyelitis of the distal femur caused by direct inoculation of Myceliophthora thermophila via a pitchfork injury to his knee. The patient demonstrated severe destructive osseous and cartilaginous infection, with slow clinical improvement, requiring the prolonged use of multiple antifungal agents. Due to the limited number of agents to which this organism was susceptible, voriconazole therapy was instituted despite limited pharmacokinetic data in children. Prolonged therapy with terbinafine, a drug generally employed for superficial saprophytic infections of skin and nails also was utilized. This case demonstrates the difficulties that can be encountered in identifying and treating this unusual but aggressive fungal organism.

## Case report

A 4-1/2 year old boy presented with a swollen right knee after being impaled in that area by a pitchfork. The pitchfork was observed to be contaminated with cow manure and hay. The knee was washed with soap and water. The following morning the knee was swollen and the boy was treated with orally administered antibiotics.

The mobility of the knee progressively decreased, and four days later the child was admitted to the hospital. Bacterial cultures of joint fluid yielded Bacillus and Enterococcus species. After a brief course of intravenous antibiotic therapy, the boy was discharged to continue orally administered antibiotics. At home, he developed increasing knee pain with inability to walk. A magnetic resonance image (MRI) obtained at transfer to our institution was consistent with infection involving the synovium, the medial femoral condyle and adjacent articular cartilage. Intravenous antibacterial therapy was instituted with vancomycin, piperacillin-tazobactam and amikacin.

Twenty seven days after the initial pitchfork injury, the patient was returned to the operating room because of persistent leg and knee swelling as well as increasing elevation of inflammatory markers with an ESR of >100 and a CRP of 6.5. An MRI revealed apparent osteomyelitis of the medial femoral condyle. New bone cultures were obtained which grew what was initially identified as a dermatophyte. Orally administered terbinafine, 125 mg (6.7 mg/kg) daily, was initiated, and the patient began to improve clinically. His CRP declined to a nadir of 1.8 with absence of fever and better movement of his leg. However, 45 days following the initial injury and 14 days after wound closure, an elevation in the CRP to 2.6, as well as an increase of purulent drainage from the knee prompted another surgical exploration of the distal femur and the addition of intravenous Ambisome, 90 mg (4.8 mg/kg) daily. At surgery, progressive bone loss was noted as well as necrosis of knee cartilage. Fungal organisms with irregular branching hyphae were noted throughout the excised cartilage, and fungus was recovered in culture two weeks later.

**Figure 1:**
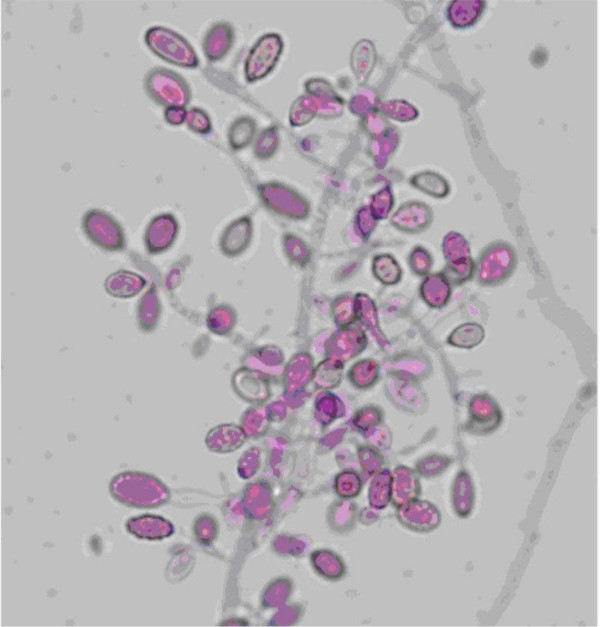
Conidia of *Myceliophthora thermophila *being produced from ampulliform swellings. Note both smooth, hyaline, immature conidia, and darker, more mature roughened conidia. Lactofuschin stain, approximately 1000×.

Meanwhile, the fungal agent that had been isolated previously was forwarded to the Fungus Testing Laboratory at the University of Texas Health Science Center in San Antonio for identification and susceptibility testing and accessioned into their stock collection as UTHSC 05-3365. There, the organism was identified as Myceliophthora thermophila, based upon observation of: 1) tan to brown powdery colonies with ill-defined margins when grown on potato flakes agar at 42°C; 2) more luxuriant growth at elevated temperatures of 35°C and 42°C than at 26°C; 3) septate vegetative hyphae with conidial production from ampulliform swellings; and 4) obovoid (inverted egg shaped) or pyriform (pear-shaped) conidia measuring 4.5–11.0 × 3.0–4.5 μm that were hyaline and smooth when immature, becoming darker and roughened at maturity (Figure 1). Antifungal susceptibility testing was performed according to the Clinical Laboratory Standards Institute (CLSI) M38-A document for filamentous fungi [2]. Although standardized susceptibility breakpoints have not been established for this organism, the isolate appeared resistant to a variety of standard antifungal agents (Table 1). Based upon these susceptibilities, voriconazole (4 mg/kg/12 hrs after a loading dose of 6 mg/kg/12 hrs for 24 hours) was added to the patient's antifungal regimen, and Ambisome was discontinued. Subsequently, the patient underwent numerous debridement procedures with gradual improvement in clinical picture and CRP. A series of progressive increases in voriconazole dosage was required based upon periodic pharmacokinetic studies to achieve appropriate blood levels (Table 2).

**Table 1:** Susceptibilities of *Myceliophthora thermophila *isolate

Drug	MIC
Amphotericin B	2 resistant*
Fluconazole	8 resistant
Itraconazole	0.125 susceptible
Voriconazole	0.06 susceptible
Caspofungin	4 resistant
Terbinafine	1 susceptible
Griseofluvin	>16 resistant

*There are no published breakpoints for this organism against any of the antifungal agents tested. Interpretations are based upon normally achievable concentrations of the drug using standard dosing regimens.

**Table 2:** Voriconazole plasma concentrations (body weight 18.6 kg)*

Voriconazole Therapy Day	Dose (mg) given every 12 hr	Dose (mg/kg)	Doses prior to kinetics	Peak (mcg/ml)	Trough (mcg/ml)
6	75 IV	4	10	0.94^a^	< 0.2
14	108 IV	5.8	8	0.6	< 0.2
24	175 IV	9.4	6	3.04	< 0.2
34	250 PO	13.4	8	2.8^b^	0.3
43	250 PO	13.4	26	5.35^c^	0.3
57	250 PO	13.4	54	2.12^b^	0.2

*Patient also receiving terbinafine 6.7 mg/kg/day

^a^IV peak @ 50 minutes post infusion

^b ^PO peak @ 2–3 hours post ingestion

^c ^PO peak @ <2 hours post ingestion

After his CRP had normalized, the patient was discharged home receiving voriconazole, 250 mg (13.4 mg/kg) orally, every 12 hours, and terbinafine 125 mg (6.7 mg/kg) orally once daily. Anti-fungal therapy is to be continued for a total of one year. The leg wound has healed, but the patient has had significant bone loss in his distal femur with involvement of the growth plate, as well as damage to the articular cartilage of the knee.

## Discussion

Myceliophthora thermophila is a melanized filamentous hyphomycete that initially grows as a white cottony colony and subsequently turns pale brown and becomes granular on a variety of media recommended for mould identification, such as potato dextrose or 2% malt agar. Its optimal growth is at 30–36°C. However, it also grows well at 42°C, with maximal growth near 50°C. Thus it is considered a thermophilic organism. Myceliophthora thermophila is found in dry pasture soil, birch chips, wood pulp, and straw compost [1]. Its cell wall contains melanin resulting in dark pigmentation, and it is considered one of the etiologic agents of phaeohyphomycosis. Phaeohyphomycosis includes those conditions in which the pathogenic mould forms fungal elements which contain melanin within their cell walls [3]. Despite the presence of melanin, cell walls of phaeoid moulds may appear hyaline or clear upon routine microscopy. Hyphal elements usually demonstrate pigment when stained with Masson-Fontana melanin stain, allowing identification of a dark fungus [4].

A recent review reports that the number of publications related to phaeohyphomycotic infections in the 1990's numbered only 150 [5]. Phaeohyphomycosis most commonly manifests as a cutaneous infection, but deep infections with invasion of the sinuses, lungs, brain, blood, and bone have also been reported [5]. Disseminated disease was reviewed by Revankar et al. who found 72 cases reported between 1966 and 2001 [6]. Notably, the majority of cases involving disseminated phaeohyphomycosis were in immune-compromised patients. The mortality rate in these individuals was high and many isolates were resistant to amphotericin B. In immunocompetent patients, most infections were associated with direct inoculation of the organism from an environmentally contaminated source.

There are just three previously reported cases of phaeohyphomycosis caused by Myceliophthora thermophila (Table 3). In the first two patients, the source of the Myceliophthora thermophila was uncertain, and both patients died despite standard anti-fungal therapy with amphotericin B. The most recently reported case of infection with this organism involved a 21-month-old boy who sustained a penetrating head injury. A brain abscess developed from which both Clostridium perfringens and Myceliophthora thermophila were isolated. The patient was treated successfully with enbloc resection of the lesion, six weeks of amphotericin B, and four months of itraconazole [9].

**Table 3:** Prior Case Reports of Myceliophthora thermophila infection

Age, Sex	History	Sites of Infection	Therapy	Outcome
7 years, M^7^	AML with neutropenia	Blood, lungs, heart	Amphotericin B	Death
22 years, F^8^	Status post Cardiovascular Surgery	Blood, aorta, heart	Amphotericin B 5-fluorocytosine	Death
21 months, M^9^	Penetrating head injury from a rusty nail in a barnyard. Brain abscess with M.thermophilia and Clostridium perfringens	Brain abscess	Amphotericin B Itraconazole	Survived

Despite convincing evidence of progressive infection in each of the three previously reported cases, it was not until well into the clinical course or even after death that identification of the etiologic agent was confirmed. It is unknown why recovery of Myceliophthora thermophila from clinical specimens is so difficult. However, the situation may be analogous to mycotic infections with more common agents such as Aspergillus, in which microbiologic isolation of the etiologic agent from grossly infected tissue can be difficult. The identification of Myceliophthorathermophila, once recovered, is also problematic, as most microbiology laboratories lack experience with this organism.

In our patient, despite evidence of ongoing infection, both operatively and preoperatively, only two samples of debrided bone or cartilage yielded Myceliophthora thermophila, despite numerous cultures of infected surgical specimens in which fungal elements could be seen histologically. Given the thermophilic nature of this organism, incubation of inoculated media at elevated temperatures may enhance recovery. Cultures of most clinical specimens are incubated at 30°C, but such a temperature would be less than optimal for growth of Myceliophthora thermophila.

Because our patient's positive cultures was obtained after approximately 2 weeks of terbinafine therapy and the organism was found to be resistant to amphotericin B, voriconazole was added to terbinafine therapy. Terbinafine is a broad-spectrum allylamine with fungicidal activity against dermatophyte species, Aspergillus species, Sporothrix schenckii, Blastomyces dermatitidis, Histoplasma capsulatum, Cryptococcus neoformans, Malassezia furfur and other important fungi. It shows in vitro synergism with amphotericin or triazoles and has been effective in combination therapy in individual patients [10-14]. It has been administered safely in a large number of children, and at high doses or for up to 12 months for invasive mycoses [15].

Voriconazole is a potent antifungal agent effective against a number of pathogens, including Aspergillus, Cryptococcus, and Candida species. It also has excellent oral bioavailability and a low rate of adverse effects [16, 17]. However, it is not approved by the Food and Drug Administration for use in children, and the appropriate dose for pediatric patients is not known. Recommendations in authoritative sources suggest the same intravenous weight-based dosages in children and adults: a loading dose of 6 mg/kg/dose every 12 hours × 1 day and a maintenance dose of 4 mg/kg/dose every 12 hours. Oral dosage is suggested at 100 mg every 12 hours for patients less than 40 kg, and 200 mg every 12 hours for patients more than 40 kg [18].

Recent investigations by Walsh et al. demonstrated that pediatric patients have a much higher rate of elimination of voriconazole per unit of body weight than do adults. Thus, children may require higher dosages to achieve blood levels consistent with adults treated at a dosage of 3–4 mg/kg [16, 17]. Additionally, the elimination of voriconazole from the blood in children appears linear when doses of 3 mg/kg to 5 mg/kg are administered every 12 hours. This is in distinction to elimination of similar doses in adults, which is non-linear or saturable. Although the exact relationship between the plasma concentration of voriconazole and the drug's clinical effectiveness is uncertain, infected adults improve at doses achieving recommended plasma concentrations. Therefore, the goal in our patient was to achieve voriconazole blood levels similar to those achieved in adults. Assuming linear pharmacokinetics, it was suggested by Walsh that a pediatric dosage of up to 11 mg/kg twice a day might be necessary to achieve drug levels equivalent to those seen in adult patients receiving 4 mg/kg of the agent every 12 hours [17]. We increased the dose of voriconazole in our patient, in stepwise fashion based upon measurements of C_max_, from 4 mg/kg to 13.4 mg/kg every 12 hours, reaching a voriconazole serum peak levels in the range of 2–5 mcg/ml, equivalent to the typical adult given only 3–4 mg/kg every 12 hours. Kinetics of voriconazole in our patient were potentially affected by concurrent administration of terbinafine.

The current case demonstrates the vigilance required in patients with traumatically induced osteomyelitis, particularly when related to direct implantation from a grossly contaminated source. Relapse of apparently appropriately treated infection while on therapy demands reassessment to be certain that an unusual or emergent microorganism is not present within the depths of the wound. In the case of a contaminated farm implement, the possibility of recovery of an unusual agent like Myceliophthora thermophila is high, potentially requiring the usage of antimicrobial agents for which there is scant pharmacologic data in children. Clinicians should be careful to obtain appropriate pharmacologic studies in order to be assured that the appropriate doses of such drugs are employed.

## Authors' contributions

LD conceived the report and helped in its writing. DS oversaw antimicrobial susceptibility testing and voriconazole blood level determinations. AH performed voriconazole pharmacokinetic analysis. PH provided clinical care and editorial assistance. JT provided surgical care and the tissue specimens from which the pathogen was recovered. RW provided clinical care and conceived the antimicrobial regimen for this patient. MC provided clinical care, editorial support and helped conceive this paper.
